# Multimodal representation learning for predicting molecule–disease relations

**DOI:** 10.1093/bioinformatics/btad085

**Published:** 2023-02-10

**Authors:** Jun Wen, Xiang Zhang, Everett Rush, Vidul A Panickan, Xingyu Li, Tianrun Cai, Doudou Zhou, Yuk-Lam Ho, Lauren Costa, Edmon Begoli, Chuan Hong, J Michael Gaziano, Kelly Cho, Junwei Lu, Katherine P Liao, Marinka Zitnik, Tianxi Cai

**Affiliations:** Department of Biomedical Informatics, Harvard Medical School, Boston, MA 02115, USA; VA Boston Healthcare System, Boston, MA 02130, USA; Department of Biomedical Informatics, Harvard Medical School, Boston, MA 02115, USA; Department of Energy, Oak Ridge National Laboratory, Oak Ridge, TN 37831, USA; Department of Biomedical Informatics, Harvard Medical School, Boston, MA 02115, USA; VA Boston Healthcare System, Boston, MA 02130, USA; Department of Biomedical Informatics, Harvard Medical School, Boston, MA 02115, USA; VA Boston Healthcare System, Boston, MA 02130, USA; Mass General Brigham, Boston, MA 02130, USA; Department of Statistics, University of California, Davis, CA 95616, USA; VA Boston Healthcare System, Boston, MA 02130, USA; VA Boston Healthcare System, Boston, MA 02130, USA; Department of Energy, Oak Ridge National Laboratory, Oak Ridge, TN 37831, USA; VA Boston Healthcare System, Boston, MA 02130, USA; Department of Biostatistics and Bioinformatics, Duke University, Durham, NC 27708, USA; Department of Biomedical Informatics, Harvard Medical School, Boston, MA 02115, USA; VA Boston Healthcare System, Boston, MA 02130, USA; Brigham and Women’s Hospital, Boston, MA 02115, USA; Department of Biomedical Informatics, Harvard Medical School, Boston, MA 02115, USA; VA Boston Healthcare System, Boston, MA 02130, USA; Brigham and Women’s Hospital, Boston, MA 02115, USA; VA Boston Healthcare System, Boston, MA 02130, USA; Department of Biostatistics, Harvard T.H. Chan School of Public Health, Boston, MA 02115, USA; Department of Biomedical Informatics, Harvard Medical School, Boston, MA 02115, USA; VA Boston Healthcare System, Boston, MA 02130, USA; Department of Biostatistics, Harvard T.H. Chan School of Public Health, Boston, MA 02115, USA; Department of Biomedical Informatics, Harvard Medical School, Boston, MA 02115, USA; Broad Institute of MIT and Harvard, Cambridge, MA 02142, USA; Harvard Data Science Initiative, Cambridge, MA 02138, USA; Department of Biomedical Informatics, Harvard Medical School, Boston, MA 02115, USA; VA Boston Healthcare System, Boston, MA 02130, USA; Mass General Brigham, Boston, MA 02130, USA

## Abstract

**Motivation:**

Predicting molecule–disease indications and side effects is important for drug development and pharmacovigilance. Comprehensively mining molecule–molecule, molecule–disease and disease–disease semantic dependencies can potentially improve prediction performance.

**Methods:**

We introduce a Multi-Modal REpresentation Mapping Approach to Predicting molecular-disease relations (M2REMAP) by incorporating clinical semantics learned from electronic health records (EHR) of 12.6 million patients. Specifically, M2REMAP first learns a multimodal molecule representation that synthesizes chemical property and clinical semantic information by mapping molecule chemicals via a deep neural network onto the clinical semantic embedding space shared by drugs, diseases and other common clinical concepts. To infer molecule–disease relations, M2REMAP combines multimodal molecule representation and disease semantic embedding to jointly infer indications and side effects.

**Results:**

We extensively evaluate M2REMAP on molecule indications, side effects and interactions. Results show that incorporating EHR embeddings improves performance significantly, for example, attaining an improvement over the baseline models by 23.6% in PRC-AUC on indications and 23.9% on side effects. Further, M2REMAP overcomes the limitation of existing methods and effectively predicts drugs for novel diseases and emerging pathogens.

**Availability and implementation:**

The code is available at https://github.com/celehs/M2REMAP, and prediction results are provided at https://shiny.parse-health.org/drugs-diseases-dev/.

**Supplementary information:**

Supplementary data are available at *Bioinformatics* online.

## 1 Introduction

Traditional approaches to drug discovery mainly rely on bench experiments, which can be costly and time-consuming. Bringing a new drug to the market, on average, costs about 2.6 billion dollars (DiMasi *et al.*, 2016) and takes over 12 years (Mohs and Greig, 2017). With the increasing availability of large-scale biomedical data and biological knowledge sources, including molecule, chemical and biological properties, computational approaches to predicting molecule indications or side effects hold great promise in improving the efficiency of the drug discovery process. Even for drugs approved for specific indications, computational methods can identify new indications or side effects of a single drug or from drug–drug interactions. Such information can assist in drug re-purposing and reducing the risk of adverse drug events. Since we focus on small-molecule drugs, we use ‘drugs’ and ‘molecules’ interchangeably throughout this paper.

Existing machine learning approaches to predicting drug–disease relations either rely on molecule chemical structure only (Zhou *et al.*, 2020) or further combine it with molecule biological properties (Jamal *et al.*, 2017; Lee and Chen, 2021; Liu *et al.*, 2012; Muñoz *et al.*, 2019; Zhang *et al.*, 2015) via multimodal methods. Chemical-only approach can effectively serve as a tool to virtually screen large chemical libraries and identify molecules to maximize the yield of downstream biological experiments. Multimodal approaches improve performance by further leveraging curated molecule properties, including target proteins, enzymes, pathways and phenotypic indications (Liu *et al.*, 2012; Muñoz *et al.*, 2019; Zhang *et al.*, 2015; Zitnik *et al.*, 2018). However, because chemical and biological properties do not contain clinical semantic information, existing methods do not comprehensively exploit drug–drug, disease–disease and drug–disease semantic dependencies. Drug–drug semantic dependency can be helpful for prediction since close molecules tend to treat or cause similar diseases (Muñoz *et al.*, 2019; Zhang *et al.*, 2016). For example, ‘statin’ drugs such as ‘atorvastatin’ and ‘fluvastatin’ are therapeutic in lowering cholesterol and the risk of cardiovascular diseases. Disease–disease dependency promotes semantically close diseases to be correlated as side effects or indications. For example, a drug that causes the side effect of ‘depression’ may also cause ‘anxiety’. Drug–disease dependency can serve as prior knowledge on a class of drugs and diseases, such as the high correlation between chemotherapeutic molecules and the side effect of ‘alopecia’. In this study, we hypothesize that effectively integrating such semantic dependency information with the chemical or biological properties can further improve the prediction.

We aim to improve the prediction of molecule–disease relations by exploiting clinical semantic information learned from electronic health records (EHR). Previous studies have suggested that semantic relationships between clinical concepts, including drugs and diseases, can be captured in an embedding vector space derived from concept co-occurrence patterns extracted from large-scale EHR data (Beam *et al.*, 2019; Hong *et al.*, 2021a). Clinical knowledge drawn from EHR data can augment existing knowledge on drug–disease relationships and drug chemical or biological properties. To synthesize information from molecule chemicals and EHR clinical semantics, we propose a Multi-Modal REpresentation Mapping Approach to Predicting molecular-disease relation (M2REMAP).

Specifically, M2REMAP first learns from large-scale EHR data the clinical semantic embedding vectors of five broad categories of codified and narrative medical concepts, i.e. drug prescription codes, diagnostic billing codes, laboratory codes, procedure codes, as well as large scale clinical concept unique identifiers (CUIs) of the Unified Medical Language System (UMLS) extracted from free-text narrative notes via natural language processing (NLP). The NLP concepts cover a broad range of semantic types, including diseases, symptoms, clinical attributes and findings, and are particularly helpful in capturing drug side effects which are often symptoms and not well coded. For example, nausea is a side effect of many cancer treatments, but it is unlikely for cancer patients to receive a diagnostic code of nausea due to treatment side effects. In the second step, M2REMAP uses a deep neural network to train a multimodal molecule representation that fuses molecule chemicals and clinical semantics. Finally, M2REMAP infers molecule–disease relations by training a relation predictor that combines the molecule representation and disease semantic embedding to jointly learn indications and side effects.

M2REMAP extends its generalization to novel diseases and novel molecules. This is achieved by performing a distribution matching of embedding vectors between large-scale molecules and EHR concepts. By representing molecules, indications, side effects, and varied clinical concepts in the same semantic embedding space, M2REMAP achieves improved generalization and high label efficiency across molecules and diseases, outperforming state-of-the-art approaches by 30.2% in PRC-AUC for side effect prediction. In addition, M2REMAP is a general molecule–disease relation predictor and addresses a critical unmet need, i.e. to effectively predict potential therapeutic molecules for novel diseases such as COVID-19, which do not have any annotated data for training.

## 2 Materials and methods

### 2.1 Overview

**Fig. 1.**
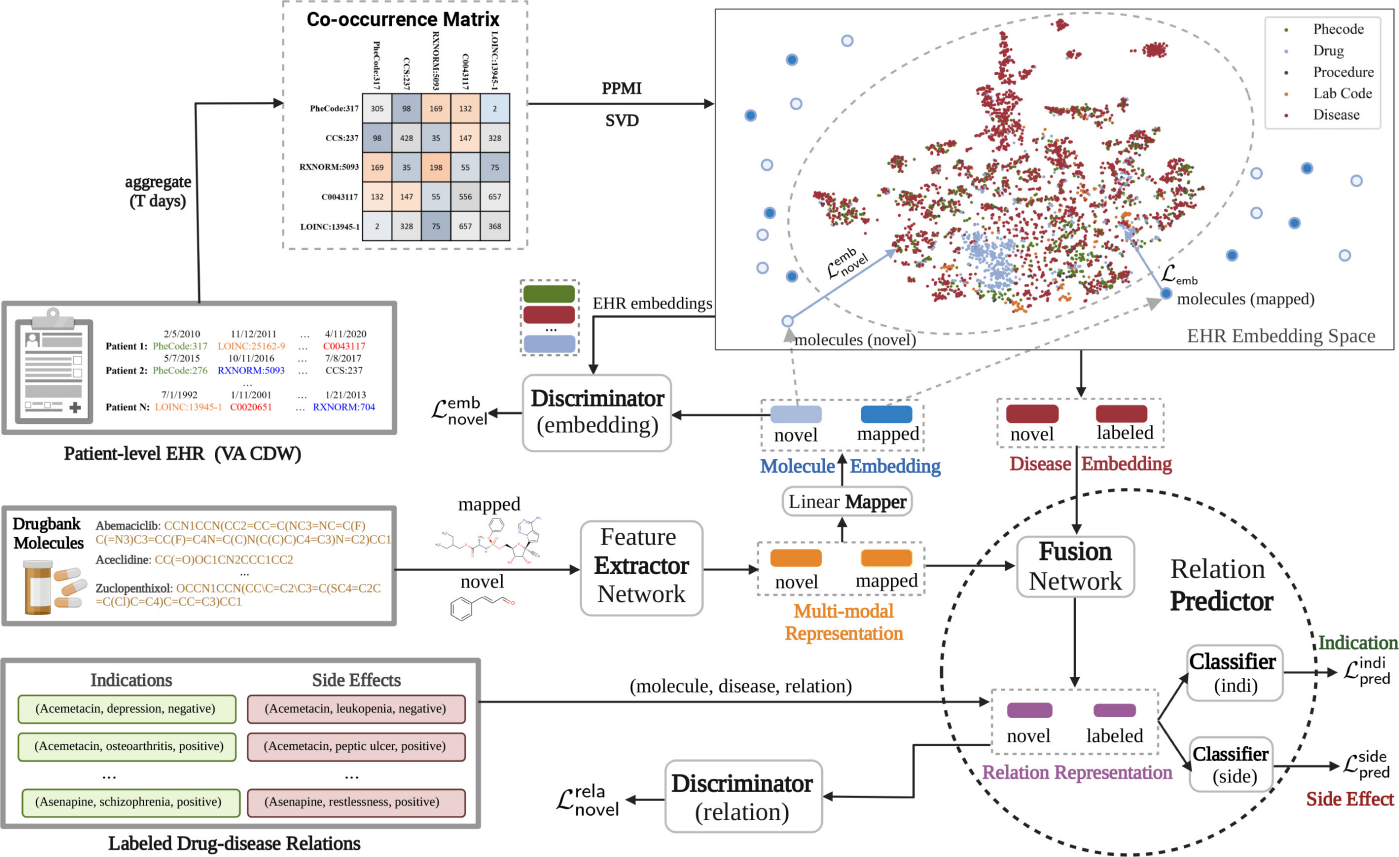
Overview of M2REMAP. By learning clinical semantic embeddings from EHR data, M2REMAP synthesized molecule chemicals and EHR semantics to attain multimodal molecule representation combined with disease EHR semantics to jointly infer indications and side effects

M2REMAP infers molecule-disease relations based on molecule chemical structures and disease clinical embeddings. As outlined in Figure 1, it consists of two steps: (i) *Clinical embedding learning* in which we learn semantic embeddings of clinical concepts from EHR data and transform large-scale molecule chemicals to the embedding space using a deep neural network; and (ii) *Molecule–disease relation prediction* in which we train a predictor network to infer molecule–disease relations by combining molecule chemicals and disease EHR embeddings. The key steps of M2REMAP are summarized in Supplementary Algorithm S1.

### 2.2 Clinical semantic learning

#### 2.2.1 EHR data

We learn semantic embedding of clinical concepts using EHR data between 1999 and 2019 from the Department of Veterans Affairs (VA) Corporate Data Warehouse (CDW), containing data from over 150 VA facilities. We include all inpatient and outpatient data from 12.6 million patients who had at least one visit. Codes occurring multiple times for the same patient within the same day are counted once per day. We include five types of clinical concepts, (i) four broad classes of codified elements, namely drugs, diagnosis, procedures and lab measurements; as well as (ii) CUIs extracted from unstructured clinical notes via the NILE software (Yu *et al.*, 2013), restricting to a subset of clinically relevant semantic types. As in Hong *et al.* (2021a), all drugs are aggregated at the ingredient level and mapped to RxNorm codes; diagnostic codes are mapped to PheCode; and procedure codes are mapped to the clinical classification software (CCS) categories (https://www.hcup-us.ahrq.gov/toolssoftware/ccs_svcsproc/ccssvcproc.jsp).

#### 2.2.2 Semantic embedding learning from EHR

We obtain clinical semantic embeddings for EHR concepts based on the matrix factorization variant of the skip-gram algorithm, which only requires a pairwise co-occurrence matrix of the concepts as the input (Levy and Goldberg, 2014). Specifically, we count co-occurrences between any pair of clinical concepts within a 30-day window of each patient and aggregate across all patients. To ensure embedding quality, we remove co-occurrence pairs with a frequency below 5000. This results in 138 193 entities, with 9211 codified concepts and 128 982 CUIs. From the co-occurrence matrix, we compute the shifted positive pointwise mutual information (SPPMI) matrix and then obtain 500-dimensional semantic embedding vectors by performing a singular value decomposition (SVD) on the SPPMI matrix. The semantic relationship of EHR concepts is well encoded in the learned embedding space, as shown previously (Beam *et al.*, 2019; Hong *et al.*, 2021a).

#### 2.2.3 Molecule embedding learning

We next map molecule chemical structures onto the EHR semantic embedding space via a supervised deep neural network. The molecules with the corresponding EHR semantic embedding are denoted as ‘mapped’ and the rest as ‘unmapped’. Based on those ‘mapped’ molecules, we train a supervised deep neural network that maps their chemical structures onto semantic embeddings, minimizing the *L*_2_ distance between the EHR embedding of the molecule and the mapped embedding.

To map the molecule structure to the space of EHR embeddings, the molecule-embedding mapping module consists of a Feature Extractor network (**E**) that transforms molecule chemical structures into multimodal representation and a Linear Mapper (**M**) that distills semantic embeddings from molecule representation. The feature extractor receives molecular SMILES as inputs and mainly consists of an embedding layer, 1D convolutional operations to capture the local interactions between tokens, and a bi-directional GRUs layer to capture the long-range sequential dependency. The CNN+bi-GRU architecture has performed well in other molecular computational tasks (Gómez-Bombarelli *et al.*, 2018; Zagidullin *et al.*, 2021). We discuss alternative architectures for this step in the discussion section. We minimize the mean squared prediction error

(1) $$L_{\mathrm{emb}}=E_{x_{i}∼D_{\mathrm{mapped}}}{\left(M(E(x_{i}))-e_{x_{i}}\right)}^{2},$$

to train **E** and **M** simultaneously, where *x_i_* is from $D_{\mathrm{mapped}}$ which consists of ‘mapped’ molecules and $e_{x_{i}}$ denotes its semantic embedding vector.

We then employ transfer learning to generalize the embedding learning to “unmapped” or novel molecules not included in EHR. Specifically, we additionally encourage the embeddings of large-scale molecule chemicals from the Drugbank (Wishart *et al.*, 2018) to be encoded by **E** onto the same semantic embedding space. This is achieved by requiring “unmapped” molecules to follow the same embedding distribution as those EHR medical concepts. To this end, the feature extractor **E** is optimized with an additional objective $L_{un}$ to measure the embedding distribution discrepancy between Drugbank molecules and EHR clinical concepts. Motivated by adversarial transfer learning, we train an additional discriminator network $D_{\mathrm{emb}}$ to distinguish the EHR embeddings from the molecule embeddings and quantify their distribution discrepancy. The **E**, together with **M**, is then trained to transform novel molecules into semantic embeddings with distributions similar to the EHR concepts. Such a *minmax* learning procedure is formulated as:

(2) $$\begin{array}{c} \underset{E,M}{\min}\underset{D_{\mathrm{emb}}}{\max}L_{\mathrm{novel}}^{\mathrm{emb}}=E_{x_{i}∼D_{db}}\;\text{log}[D_{\mathrm{emb}}(M(E(x_{i})))]\\ +E_{e_{j}∼D_{\mathrm{ehr}}}\;\text{log}[1-D_{\mathrm{emb}}(e_{j})], \end{array}$$

where *x_i_* denotes a molecule chemical structure from Drugbank *D_db_* and *e_j_* is a semantic embedding from $D_{\mathrm{ehr}}$ which consists of all clinical concepts. We use a multi-layer perception network as the discriminator $D_{\mathrm{emb}}$ parameterized by $\theta_{D_{\mathrm{emb}}}$.

### 2.3 Molecule–disease relation prediction

#### 2.3.1 Multimodal molecule representation

To optimize the prediction of molecule–disease relations, M2REMAP further fine-tunes the representation of molecules based on observed labels on the known relationships. To this end, the feature extractor **E** is further refined in a multi-task manner to minimize the two aforementioned embedding learning losses $L_{\mathrm{emb}}$ in (1) and $L_{\mathrm{novel}}^{\mathrm{emb}}$ in (2) and also the binary cross-entropy prediction loss:

(3) $$\begin{array}{c} L_{\mathrm{pred}}=-E_{(x_{i},d_{i},y_{i})∼D_{\mathrm{label}}}(y_{i}*\;\text{log}(P(E(x_{i}),e_{d_{i}}))\\ -(1-y_{i})*\;\text{log}(1-P(E(x_{i}),e_{d_{i}}))), \end{array}$$

where *i* indexes the relationship pair with label *y_i_* sampled from annotated molecule–disease dataset $D_{\mathrm{label}}$, and $e_{d_{i}}$ denotes the embedding of disease *d_i_* from $D_{\mathrm{ehr}}$. We next detail the construction of the relation predictor **P**, which takes separate forms for indications versus side effects.

#### 2.3.2 Relation learning

To comprehensively capture drug–drug, disease–disease and drug–disease semantic dependencies, M2REMAP infers general molecule–disease relations by sharing the pairwise relation predictor **P** across multiple diseases and drugs. To achieve this, **P** combines multimodal molecule representation and disease semantics and is trained to learn invariant relation representations across novel molecule–disease combinations of novel molecules or diseases, as shown in Figure 1. Therefore, **P** is additionally optimized by $L_{\mathrm{novel}}^{\mathrm{rela}}$:

(4) $$\begin{array}{c} \underset{P}{\min}\underset{D_{\mathrm{rela}}}{\max}L_{\mathrm{novel}}^{\mathrm{rela}}=E_{x_{i}∼D_{db},d_{i}∼D_{\mathrm{ehr}}}\;\text{log}[D_{\mathrm{rela}}(P(E(x_{i}),e_{d_{i}}))]\\ +E_{(x_{j},d_{j})∼D_{\mathrm{label}}}\;\text{log}[1-D_{\mathrm{rela}}(P(E(x_{j}),e_{d_{j}}))], \end{array}$$

where $D_{\mathrm{rela}}$, with the same architecture as the $D_{\mathrm{emb}}$, is a discriminator network trained to distinguish the relation representation of annotated molecule–disease combinations from those of novel combinations. Molecule *x_j_* and *d_j_*, with embedding $e_{d_{j}}$, are from the annotated molecule–disease data $D_{\mathrm{label}}$.

Integrating all steps, the M2REMAP model is trained with a joint objective $L_{\mathrm{joint}}$, which is formulated as:

(5) $$L_{\mathrm{joint}}=L_{\mathrm{pred}}^{\mathrm{indi}}+L_{\mathrm{pred}}^{\mathrm{side}}+\beta L_{\mathrm{emb}}+\gamma L_{\mathrm{novel}}^{\mathrm{emb}}+\delta L_{\mathrm{novel}}^{\mathrm{rela}},$$

where $L_{\mathrm{pred}}^{\mathrm{indi}}$ and $L_{\mathrm{pred}}^{\mathrm{side}}$ denote the prediction loss, defined in Equation (3), of indication and side effects, respectively. *β*, *γ* and *δ* are hyper-parameters that balance the multiple objectives. When minimizing $L_{\mathrm{joint}}$, some nuisance parameters should be optimized using specific components. For example, the embedding mapper **M** can be optimized only using $L_{\mathrm{emb}}$. The hyper-parameter selection is detailed in Supplementary Note S6.

#### 2.3.3 Semantic-guided sampling of negative drug–disease relations

Each drug is typically reported to have some indications or side effects, often noted in the widely used SIDER (Liu *et al.*, 2012) and FDA reports (Zhang *et al.*, 2021), which constitute the positive drug–disease pairs. However, validated annotations on negative drug–disease relationship pairs are scarce. Most existing methods treat the unreported drug–disease pairs as the negatives (Muñoz *et al.*, 2019; Yamanishi *et al.*, 2012; Zhang *et al.*, 2016). This could be problematic and result in false negatives due to incomplete annotations, causing bias in both training and evaluation. To alleviate this issue, M2REMAP guides the selection of negative molecule–disease relations by exploiting the semantic similarity as prior knowledge. Specifically, for each drug, we require the negative indications or side effects to be semantically dissimilar to all of the drug’s reported or annotated indications or side effects. In this case, for a drug with the partially annotated side effect of ‘C0011603 (dermatitides)’, the side effects of ‘C0037274 (dermatosis)’ and ‘C0023530 (itch)’, which co-occur with ‘C0011603 (dermatitides)’ frequently as side effects, would not be incorrectly treated as negatives. To achieve this, we measure the disease closeness using the semantic cosine similarity and remove the diseases from the negatives with high cosine similarity to the annotated diseases.

In addition, we also study using validated negative drug side effects pairs for extra validations by creating a small dataset based on literature reviews of clinical-trials meta-analysis results (Edwards *et al.*, 1999; Fioravanti *et al.*, 2014; Golder *et al.*, 2011), which is provided in Supplementary Note S7.

## 3 Experiments

We validate M2REMAP in three parts. (i) We visualize the learned EHR clinical semantic embedding vectors and show that it captures drug–disease semantic relationship; (ii) we numerically evaluate M2REMAP in learning molecule indications and side effects using annotated datasets; (iii) we investigate the novel prediction of molecule–disease relations.

### 3.1 EHR embedding visualization

#### 3.1.1 Settings

**Fig. 2:**
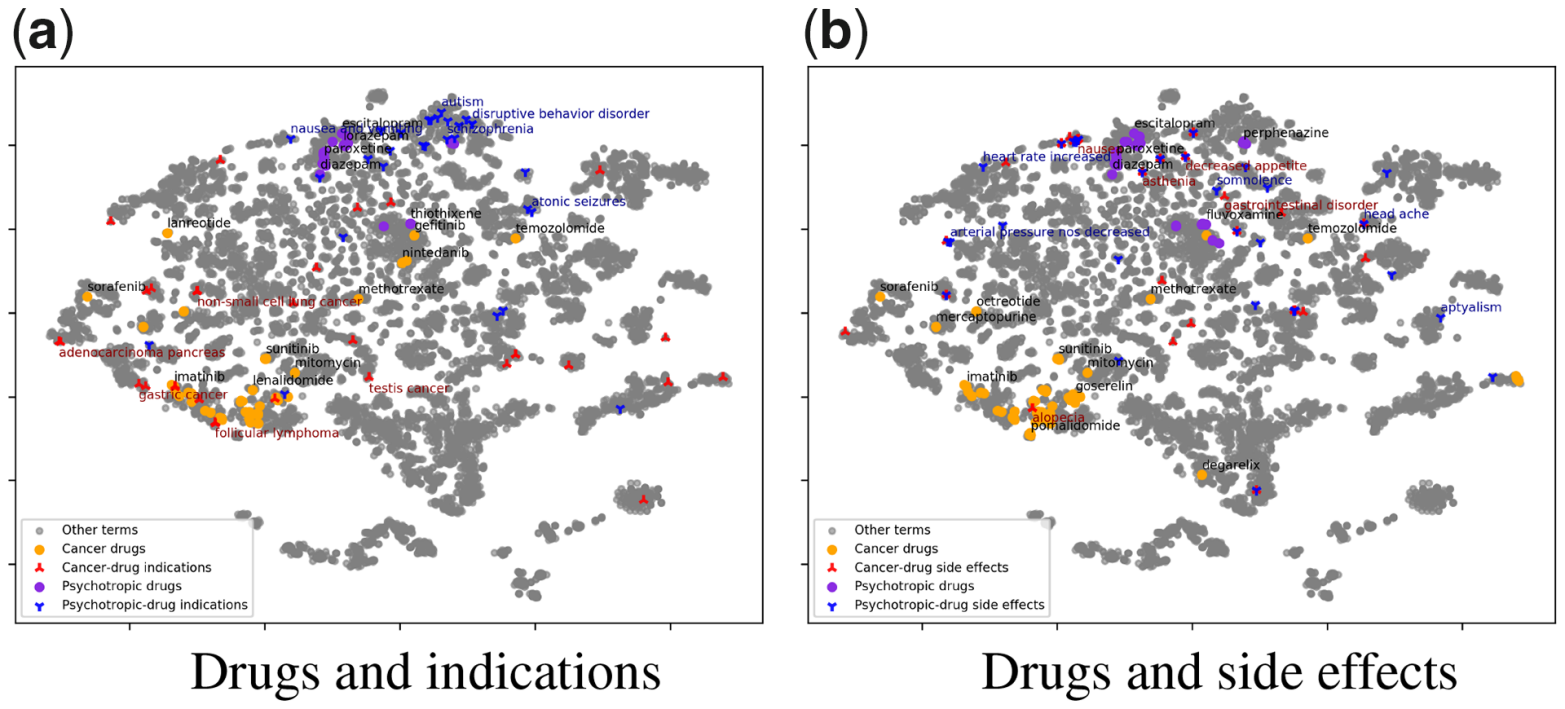
Drug–disease embedding visualization. We visualize the EHR semantic embedding of cancer and psychotropic drugs and their reported indications and side effects. (**a**) drugs and indications; (**b**) drugs and side effects

We first examine the relationships between drugs and diseases by visualizing the EHR embedding space. The embedding vectors are first reduced to two dimensions via PCA and then visualized using t-SNE (Van der Maaten and Hinton, 2008). We visualize 46 cancer drugs (https://www.cancer.gov/about-cancer/treatment/drugs) and 26 psychotropic drugs (https://www.healthpartners.com/ucm/groups/public/@hp/@public/documents/documents/entry_194823.pdf), which are commonly used in clinical settings, and their related top-30 indications reported on PrimeKG dataset (Chandak *et al.*, 2022) in Figure 2a and their top-30 reported side effects on the SIDER 4.1 dataset (Kuhn *et al.*, 2016) in Figure 2b. Meanwhile, we also visualize all EHR concepts and Drugbank molecules in Supplementary Figure S3a, showing that they are well-aligned in the embedding space.

#### 3.1.2 Results

As shown in Figure 2a, cancer drugs generally cluster together in the semantic embedding space and are far away from the psychotropic drugs. Most of the indications are close to the corresponding drugs, and the indications of cancer drugs are distinct from those of psychotropic drugs in the embedding space. That is, indications are an important indicator of drug semantics, which explains why observing indications considerably improves the learning of drug side effects in (Liu *et al.*, 2012; Zhang *et al.*, 2015). A few drugs indicated for cancers of specific organs are far from the majority in the embedding space. For example, ‘Sorafenib’ is used for ‘Renal cell carcinoma’, and ‘Temozolomide’ is used for brain cancer and is reasonably close to psychotropic drugs in the embedding space.

The semantic dependencies between drugs and side effects are more complex than those between drugs and indications. Side effects generally do not cluster together with the corresponding medications in the embedding space, as shown in Figure 2b. This is because, in contrast to the high co-occurrence between indications and the corresponding drugs in EHR data, side effects occur less frequently and hence have fewer co-occurrences with the corresponding drugs. In addition, many side effects, such as ‘nausea’ and ‘decreased appetite’, are often not specific and shared across drugs.

### 3.2 Molecule indication prediction

#### 3.2.1 Settings

Based on annotated drug–disease indications, we comprehensively evaluate M2REMAP under three different settings. (i) *Cross-molecule validation* where we randomly choose 70% molecules for training, 10% for validation and 20% for evaluation, which is the default setting unless noted; (ii) *Cross-year validation* where we use the drugs reported in SIDER 4.1 (Kuhn *et al.*, 2016), marketed before 2015, for training and the drugs marketed since 2015 for evaluation; and (iii) *Cross-disease validation* in which we randomly select 70%, 10% and 20% diseases for training, validation and evaluation, respectively. Cross-year validation aims to evaluate model generalization to predict newly marketed drugs with already on-market drugs as training. Cross-disease validation test model ability to generalize to novel diseases not encountered by the model during training.

We experiment on PrimeKG (Chandak *et al.*, 2022), which includes annotated drug-indication relations and SIDER 4.1 (Kuhn *et al.*, 2016), which consists of reported side effects for joint training. There are 349 indications included that are observed by at least three drugs in PrimeKG. Both datasets are detailed in Table 1. Instead of using the raw 500-D VA embedding, we reduce its dimensionality to 100 by performing PCA among the drugs and diseases observed in PrimeKG and SIDER 4.1. Sensitivity analysis on the embedding dimensionality is provided in Supplementary Note S9. In addition to the proposed M2REMAP, we report five variants of M2REMAP to show how different components affect the performance: (i) *M2REMAP(w/o multimodal)* in which the molecule representation is not trained to incorporate EHR semantic information, i.e. removing $L_{\mathrm{emb}}$ and $L_{\mathrm{novel}}^{\mathrm{emb}}$ from the objective function; (ii) *M2REMAP(w/o semantic)* in which no EHR semantic information is used and diseases are represented by one-hot encoding; (iii) *M2REMAP(w/o joint)* in which indications and side effects are trained separately; (iv) *M2REMAP*(*w/o* $L_{\mathrm{novel}}$) in which components for generalizing to novel molecules and diseases, $L_{\mathrm{novel}}^{\mathrm{emb}}$ and $L_{\mathrm{novel}}^{\mathrm{rela}}$, are removed; and (v) *M2REMAP(base)* denotes the baseline deep neural network without incorporating semantics and joint training. In the base model, the diseases are one-hot encoded. Still, the predictor is shared across all diseases, a practice found to achieve better performance than training each disease independently (Liu *et al.*, 2012; Muñoz *et al.*, 2019; Zhang *et al.*, 2016). Since the drug indication and drug side effects are jointly trained, diseases that are reported as indications and side effects simultaneously would be treated as indications only. To measure the performance, we report the area under the receiver operating characteristics curve (ROC-AUC), the area under the precision-recall curve (PRC-AUC), and the average precision at k (AP@k). The results are averaged over five random partitions of the training versus test data.

**Table 1.** Datasets details of the annotated drug–disease relations

Setting	Dataset	Drug	Disease	Sample
Drug indications	PrimeKG (Chandak *et al.*, 2022)	1281	349	3718
	SIDER-PrimeKG	1242:39	349	3718
Side effects(mono)	SIDER(Liu) (Liu *et al.*, 2012)	769	1228	43719
	SIDER(Zhang) (Zhang *et al.*, 2015)	747:263	2853	80177
	SIDER 4.1 (Kuhn *et al.*, 2016)	1355	2923	99159
	SIDER-FAERS(mono)	1355:159	3355	104223
Side effects(DDI)	TWOSIDES (Yu *et al.*, 2021)	645	200	54261
	FAERS2021 (Zhang *et al.*, 2021)	925	2453	70385
	SIDER-FAERS(DDI)	846:79	2453	70385

*Note*: The PrimeKG consists of drug-indication pairs. Both mono-drug and drug–drug interaction (DDI) are included for side effects.

#### 3.2.2 Results

As shown in Table 2, M2REMAP achieves a PRC-AUC of 0.649, which improves from the 0.525 of the baseline model by 23.6%. The AP@3 is 0.295, which is reasonably high considering that there are only 2.9 reported indications on average for each molecule. Compared to the LSP-ADR (Liu *et al.*, 2012) and MEDICASCY (Zhou *et al.*, 2020), performance gains of M2REMAP are considerable, outperforming state-of-the-art chemical-only model, MEDICASCY, by 5.9% in ROC-AUC and 37.5% in PRC-AUC. Without incorporating EHR embeddings, M2REMAP(w/o semantic) performs only comparably to the baseline model. Reduced version M2REMAP(w/o multimodal) performs worse than the baseline model in PRC-AUC and AP@3, which verifies the importance of multimodal molecule representation. In addition, we find that jointly learning indications and side effects helps boost performance. This is consistent with the results reported in Zhou *et al.* (2020), Zhang *et al.* (2016) and Liu *et al.* (2012). The training objective $L_{\mathrm{novel}}$ improves model generalization cross molecules and brings improvements of 5.5% in PRC-AUC, 2.0% in ROC-AUC and 6.1% in AP@3.

**Table 2.** Results of molecule indication learning on the PrimeKG dataset (Chandak *et al.*, 2022)

Methods	ROC-AUC	PRC-AUC	AP@3
LSP-ADR (Liu *et al.*, 2012)	0.821	0.439	0.123
MEDICASCY (Zhou *et al.*, 2020)	0.833	0.472	0.173
M2REMAP(base)	0.826	0.525	0.262
M2REMAP(w/o multimodal)	0.846	0.458	0.225
M2REMAP(w/o semantic)	0.849	0.517	0.268
M2REMAP(w/o joint)	0.853	0.577	0.257
M2REMAP(w/o $L_{\mathrm{novel}}$)	0.865	0.615	0.278
M2REMAP	**0.882**	**0.649**	**0.295**
M2REMAP(cross-year)	0.853	0.573	0.256
M2REMAP(cross-disease)	0.758	0.414	0.175
Random guess(cross-disease)	0.509	0.058	0.022

For the cross-disease prediction, namely, to predict the relations involving novel diseases, M2REMAP drops by 36.2% in PRC-AUC and by 14.1% in ROC-AUC over cross-molecule, which is substantially worse than the cross-year setting. One of the reasons is that M2REMAP is trained from <350 diseases, thus with weaker cross-disease generalization than over cross-molecule. Since existing methods require annotated data for predicted diseases and cannot always successfully generalize to novel diseases, M2REMAP encouragingly outperforms the random guess by 613.8% in PRC-AUC and 48.9% in ROC-AUC. In Supplementary Note S8, we provide a detailed analysis of performance gains across different drug/indication groups brought by the EHR embedding vectors. We observe that drugs from ‘musculo-skeletal system’ and ‘respiratory system’ benefit the most from the semantic embedding, while for diseases, the improvement from ‘nervous system diseases’ is the most significant, followed by ‘metabolic diseases’.

### 3.3 Side effects prediction

#### 3.3.1 Settings

For side effect prediction, we evaluate drug side effects caused by a single drug or drug interactions. The same three settings as in the molecule indication prediction are used. For mono-drug side effects, we experiment on SIDER(Liu) (Liu *et al.*, 2012), SIDER(Zhang) (Zhang *et al.*, 2015), nd SIDER-FAERS(mono) which aims for cross-year validation by learning on SIDER 4.1 (Kuhn *et al.*, 2016) with annotated drugs marketed before 2015 and evaluating on FAERS2021 (Zhang *et al.*, 2021) with newly marketed drugs since 2015. For side effects by drug–drug interactions, we evaluate on TWOSIDES (Zitnik *et al.*, 2018), following the protocols of Yu *et al.* (2021) and FAERS2021 (Zhang *et al.*, 2021). Of note, for DDIs, most existing methods (Grover and Leskovec, 2016; Yu *et al.*, 2021; Zitnik *et al.*, 2018) assume all drugs available for training and aim to learn models that predict the interaction by their new combinations. To study the generalization to novel diseases, we construct SIDER-FAERS(DDIs) based on FAERS2021 annotations, namely using drugs in SIDER 4.1 (Kuhn *et al.*, 2016) for training and the rest of the drugs in FAERS2021 for evaluation. The datasets are detailed in Table 1.

#### 3.3.2 Results

As shown in Table 3 for mono-drug side effects, M2REMAP attains substantially higher PRC-AUC compared to ISRNS (Zheng *et al.*, 2019), the best performing existing method evaluated on SIDER(Liu), and MEDICASCY (Zhou *et al.*, 2020), the SOTA model on SIDER(Zhang). M2REMAP improves the PRC-AUC from 0.542 of ISRNS to 0.626 on the SIDER(Liu), and from 0.394 of MEDICASCY to 0.513 on SIDER(Zhang). Supplementary Note S8 provides a detailed analysis of performance gains across different drug/side effect groups brought by the EHR embedding vectors. We observe that drugs from different groups consistently benefit from the semantic embedding, and the improvements from ‘respiratory system’ and ‘dermatologicals’ are the most substantial. When grouping side effects into different categories, most categories attain higher accuracy, with the ‘eye/ear diseases’ benefiting the most, followed by ‘mental disorders’.

**Table 3.** Results of predicting mono-molecule side effects

Dataset	Method	Feature	ROC-AUC	PRC-AUC
SIDER(Liu)	LSP-ADR (Liu *et al.*, 2012)	Multimodal	0.885	0.251
	FS-MLKNN (Zhang *et al.*, 2015)	Multimodal	0.903	0.480
	LNSM-CMI (Zhang *et al.*, 2016)	Multimodal	0.909	0.491
	ISRNS (Zheng *et al.*, 2019)	Multimodal	0.909	0.542
	M2REMAP(base)	Chemical	0.905	0.517
	M2REMAP	Chemical	**0.915**	**0.626**
SIDER(Zhang)	MLP (Muñoz *et al.*, 2019)	Chemical	0.894	0.355
	FS-MLKNN (Zhang *et al.*, 2015)	Chemical	0.872	0.365
	LNSM-CMI (Zhang *et al.*, 2016)	Chemical	0.885	0.380
	MEDICASCY (Zhou *et al.*, 2020)	Chemical	**0.902**	0.394
	M2REMAP(base)	Chemical	0.889	0.405
	M2REMAP	Chemical	0.901	**0.513**
SIDER-FAERS(cross-year)	M2REMAP	Chemical	0.873	0.507
SIDER 4.1(cross-disease)	M2REMAP	Chemical	0.788	0.288
	Random guess	Chemical	0.513	0.065

The results of side effects caused by drug–drug interactions are reported in Table 4. For TWOSIDES, which reports each side effect with at least 900 drug–drug combinations, we report AP@50, while for FAERS2021, which reports each side effect only with 25.9 drug pairs on average, we report AP@3. On the TWOSIDES, M2REMAP attains PRC-AUC and AP@50 of 0.978 and 0.993, respectively, which are substantially higher than the 0.934 and 0.888 of the SOTA method SumGNN (Yu *et al.*, 2021). For drug–drug interaction with novel drugs, M2REMAP is observed with significant performance drops on the SIDER-FAERS, with PRC-AUC falling by 7.9% and AP@3 by 11.9%, compared to the cross-molecule validation on FAERS2021. The results indicate that its challenging for drug–drug interaction learning to generalize to novel drugs.

**Table 4.** Results of molecule side effects caused by drug–drug interactions

Dataset	Method	Feature	ROC-AUC	PRC-AUC	AP@k
TWOSIDES	MLP (Rogers and Hahn, 2010)	Multimodal	0.826	0.812	0.735 (@50)
	Node2Vec (Grover and Leskovec, 2016)	Multimodal	0.907	0.889	0.830 (@50)
	SumGNN (Yu *et al.*, 2021)	Multimodal	0.949	0.934	0.888 (@50)
	M2REMAP(base)	Chemical	0.901	0.912	0.897 (@50)
	M2REMAP	Chemical	**0.986**	**0.978**	**0.993** (@50)
FAERS2021	M2REMAP(base)	Chemical	0.912	0.897	0.858 (@3)
	M2REMAP	Chemical	0.987	0.985	0.975 (@3)
SIDER-FAERS (cross-year)	M2REMAP(base)	Chemical	0.835	0.776	0.597 (@3)
	M2REMAP	Chemical	0.912	0.907	0.859 (@3)

### 3.4 Case studies of novel predictions

We conduct case studies to examine M2REMAP’s novel molecule–disease predictions. First, we study the molecules predicted to be therapeutic to cancers. Then, we predict potential molecules for COVID-19, a novel disease, considering available treatment information. Finally, we examine side-effect predictions of drugs recently withdrawn due to severe adverse events.

#### 3.4.1 Cancer-therapeutic molecules

We first examine the Drugbank (Wishart *et al.*, 2018) molecules that M2REMAP predicts to be potentially therapeutic to cancers. We rank the molecules based on cumulative confidence, which sums up the probability of cancer-related indications among their top 50 predictions. Among the top 20 molecules, we can find 9 with literature supports as shown in Table 5, which provides each molecule with the top two cancer-related predictions. The investigational DB03701 (Vanoxerine), which is semantically close to liver-related diseases as shown in Supplementary Figure S2b, is predicted with indications of ‘liver cell carcinoma (C2239176)’. This is supported by Zhu *et al.* (2021), which has shown its therapeutic effect on hepatocellular carcinoma. The DB14184 (Cinnamaldehyde), in an experimental stage, is predicted to treat ‘malignant tumor of breast’, which is supported by Chiang *et al.* (2019) and Liu *et al.* (2020). There are also two approved drugs. DB00715 (Paroxetine), which is predicted to treat ‘malignant tumor of breast’, is a type of antidepressant and labeled with psychotropic indications including ‘major depressive disorder’, ‘obsessive-compulsive disorder’, etc. Some recent studies have revealed its anti-cancer effects, for example, breast cancer in Cho *et al.* (2019) and colon cancer in Jang *et al.* (2019). DB02701 (Nicotinamide) is a water-soluble vitamin B3 or niacin and is labeled with indications of ‘acne vulgaris’, ‘pellagra’ and ‘niacin deficiency’. M2REMAP predicts it with indications of ‘neoplasm of prostate (C0033578)’ and ‘malignant tumor of the cervix (C0007847)’. The effectiveness of the drug as cancer chemoprevention and therapy is supported by Nikas *et al.* (2020) and Scatozza *et al.* (2020).

**Table 5.** Literature validation of Drugbank molecules predicted to be therapeutic to cancers

Molecule	M2REMAP prediction	Stage	Evidence
DB14980(AZD-6482)	Malignant lymphoma, glioblastoma multiforme	Investigational	Anti-proliferation (Zhao *et al.*, 2021)
DB14017(H3B-8800)	Hairy cell leukemia, acute myeloid leukemia	Investigational	Antitumor (Seiler *et al.*, 2018)
DB15190(GLPG-0259)	Non-small cell lung cancer, metastatic malignant melanoma	Investigational	Tumor metastasis (Wang *et al.*, 2021)
DB03701(Vanoxerine)	Medullary thyroid carcinoma, liver cell carcinoma	Investigational	Hepatocellular carcinoma (Zhu *et al.*, 2021)
DB06266(Lonidamine)	Malignant tumor of breast, medullary thyroid carcinoma	Investigational	Antitumour (Huang *et al.*, 2020)
DB11455(Robenacoxib)	Hairy cell leukemia, malignant tumor of breast	Experimental	antitumour (Nikas *et al.*, 2020)
DB14184(Cinnamaldehyde)	Malignant tumor of breast, metastatic malignant melanoma	Experimental	Breast cancer (Liu *et al.*, 2020)
DB00715(Paroxetine)	Malignant tumor of breast, gestational trophoblastic neoplasia	Approved	Anticancer activity (Cho *et al.*, 2019)
DB02701(Nicotinamide)	Neoplasm of prostate, malignant tumor of cervix	Approved	Cancer therapy (Sauer *et al.*, 2021)

#### 3.4.2 Molecules for COVID-19

We next study the generalization of M2REMAP in predicting therapeutic molecules for COVID-19 as a novel disease. To this end, we first train an embedding vector for COVID-19, which is represented by the EHR concept of ‘COVID-19 PCR test positive’, based on the skip-gram algorithm using the co-occurrence matrix of EHR concepts assembled for VA patients with a COVID-19 diagnostic code between March 2020 and September 2021. This allows us to create a new set of embeddings for 5272 EHR concepts, including COVID-19, and a subset of 2105 diagnosis codes shared with the existing VA EHR concepts. To map the COVID-19 embedding to the VA embedding space, we train a neural network in a supervised manner to learn the mapping from the new embeddings with COVID-19 to the previously trained VA embedding. Therein, we obtain the VA embedding vector of COVID-19. The detailed learning procedures are provided in Supplementary Note S2. With the newly trained COVID-19 embedding and the relation predictor, we identify the top Drugbank molecules with a high likelihood of having COVID-19 as their indications. We rank the molecules by the prediction scores given by M2REMAP. In addition, as described in Section 3.1, molecules tend to be close to the corresponding target indications. Therefore, we refine the prediction results by removing the molecules with a cosine similarity of <0.1 to COVID-19.

The results are provided in Table 6. Among the top 20 predictions by M2REMAP, which are visualized in Supplementary Figure S2, seven molecules are found with literature evidence. M2REMAP successfully predicts DB14761 (Remdesivir), which FDA approved to treat COVID-19. The top candidate predicted by M2REMAP is DB00481 (Raloxifene), a selective estrogen receptor modulator for the treatment and prevention of postmenopausal osteoporosis and cancer, which is recently shown to potentially treat SARS-CoV-2 infection in Hong *et al.* (2021b) and Allegretti *et al.* (2022). The DB01268 (Sunitinib), a kinase inhibitor, is also of great potential as an anti-coronavirus drug, as shown in Wang *et al.* (2020). Three molecules are still not marketed. Specifically, DB13527 (Proglumetacin), at an experimental stage, is shown to inhibit SARS-CoV-2 in Alves *et al.* (2021), DB12181 (Dalcetrapib), at an investigational stage, is shown to have inhibitory effects on the SARS-CoV-2 3CL protease and viral replication (Niesor *et al.*, 2021), and DB05420 (Gallium maltolate), at an investigational stage, is shown to inhibit the replication of SARS-CoV-2 (Bernstein and Zhang, 2020).

**Table 6.** Drugbank molecules predicted to be therapeutic to COVID-19

Molecule	Ranking	Stage	Evidence
DB14761(Remdesivir)	17	Approved; investigational	Approved for COVID-19
DB00481(Raloxifene)	1	Approved; investigational	Hong *et al.* (2021b)
DB01268(Sunitinib)	3	Approved; investigational	Wang *et al.* (2020)
DB05420(Gallium maltolate)	5	Investigational	Bernstein and Zhang (2020)
DB13527(Proglumetacin)	6	Experimental	Alves *et al.* (2021)
DB12181(Dalcetrapib)	13	Investigational	Niesor *et al.* (2021)
DB11753(Rifamycin)	14	Approved; investigational	Pathak *et al.* (2021)

#### 3.4.3 Drug withdrawal prediction

We examine the side effect predictions of the 12 drugs withdrawn from the market because of severe adverse effects (https://en.wikipedia.org/wiki/List_of_withdrawn_drugs) since 2008. We train M2REMAP on the SIDER 4.1 (Kuhn *et al.*, 2016). As shown in Table 7, the results are in three parts. In the first part, four drugs are not included in SIDER 4.1 and thus are considered novel molecules. M2REMAP correctly predicts the leading causes of withdrawal. The second part in Table 7 contains three drugs included in SIDER 4.1, but no side effects related to the withdrawal are reported. The three drugs, Lorcaserin, Ranitidine and Ingenol mebutate, are withdrawn because of the increased risk of cancers, which aligns with M2REMAP predictions. For those seven drugs without withdrawal-related side effects reported on the SIDER 4.1, M2REMAP achieves a recall@50 of 1.0, significantly better than a random guess of 0.141. The third part in Table 7 consists of five drugs with related side effects reported in SIDER 4.1, and M2REMAP also successfully specifies the withdrawal causes.

**Table 7.** Side-effect predictions of drugs recently withdrawn due to adverse events

Drugbank ID	M2REMAP prediction	Regions withdrawn	Reason for withdrawal
DB06623(Flupirtine)	Cholangitis, hepatotoxicity	EU, 2018	Liver injury
DB13324(Tetrazepam)	Lip ulceration, localized erythema	EU, 2013	Serious cutaneous reactions
DB01283(Lumiracoxib)	Jaundice hepatocellular, hepatocellular injury	Worldwide, 2008	Liver damage
DB09004(Clobutinol)	Bradyarrhythmia, atrial rhythm	Worldwide, 2008	Ventricular arrhythmia
DB04871(Lorcaserin)	Carcinoma testes, breast cancer	US, 2020	Risk of cancer
DB00863(Ranitidine)	Carcinoma of prostate, brain neoplasm	US, 2020	Risk of cancer
DB05013(Ingenol mebutate)	Malignant melanoma, depigmentation	EU, 2020	Risk of skin cancers
DB06268(Sitaxentan)	Jaundice hepatocellular	Germany, 2010	Hepatotoxicity
DB01105(Sibutramine)	Bradyarrhythmia, coronary heart disease,	US, 2010	Cardiovascular events
DB00647(Dextropropoxyphene)	Atrial rhythm, sinus arrest	Worldwide, 2010	Heart attacks and stroke
DB00412(Rosiglitazone)	Hypernatraemia, sinus arrest	EU, 2010	Heart attacks and death
DB06155(Rimonabant)	Dependence psychological, depressive disorder	Worldwide, 2008	Depressive disorders

## 4 Discussions

M2REMAP attains higher accuracy and generalizability for predicting molecule–disease relations over existing methods by effectively combining chemical structure, and semantic representations of clinical concepts learned from EHR. By mapping large-scale clinical concepts, including indications, side effects and drug molecules, onto a shared semantic embedding space, M2REMAP bridges the semantic gap between molecule chemicals and clinical concepts. M2REMAP is flexible and relatively robust to the choice of different pipelines for feature extractors. For example, the current implementation of the feature extractor **E** in M2REMAP uses the CNN+bi-GRU architecture, which can potentially be replaced by alternative network architectures such as the Transformer (Vaswani *et al.*, 2017) with molecular SMILE as input and MPNN (Gilmer *et al.*, 2017) with molecular graphs as input. As shown in Supplementary Note S1, M2REMAP with different network architectures has comparable performances although CNN+bi-GRU appears to be the most robust.

When integrating EHR embedding information with chemical structures and existing labels on side effects and indications, it is critical to leverage both structured and textual EHR data extracted via NLP, especially for studying side effects. Drug side effects are often symptoms that cannot be well captured by diagnostic codes but can be well represented by UMLS CUIs that cover a broader range of semantic types, including disease, symptoms, clinical attributes and findings. In addition, even for side effects that can be mapped to diagnostic codes, physicians may record the side effects information in clinical notes but only assign diagnostic codes associated with the disease the patient is treated for.

As demonstrated by the COVID-19 case study, M2REMAP enables predictions of therapeutic molecules for novel diseases by leveraging EHR as a live system to learn semantic representation for novel diseases. This is a key advantage of M2REMAP over existing methods which typically require the diseases to be pre-defined with annotated drug–disease relations. Furthermore, there is currently no effective strategy to represent novel diseases when limited knowledge exists. Neither one-hot encoding nor text description can fully capture the clinical characteristics of a novel disease. On the other hand, results from the COVID-19 case study suggest that the co-occurrence patterns of EHR concepts can effectively train embeddings to represent novel diseases, which can then be integrated into the M2REMAP pipeline for the prediction of indications.

There are several directions for future study. First, by linking with all EHR concepts, the semantic embedding can also be used to infer the relationship between molecules and other clinical concepts, for example, laboratory and therapeutic procedures, and improve molecule-related research such as predicting molecule properties, target proteins, etc. The current implementation of M2REMAP achieved robust performance by combining information from chemical structure and clinical information from EHR. However, we expect that M2REMAP can be modified to gain higher accuracy by further leveraging molecule biological properties such as target proteins, enzymes, pathways, etc., which are available for a subset of molecules via semi-supervised learning.

## Supplementary Material

btad085_Supplementary_Data

## Data Availability

The data underlying this article are available in the article and in its online supplementary material.
