# Supplementary material for: Multimodal representation learning for predicting molecule–disease relations
Source: Bioinformatics. 2023 Feb 10;39(2):btad085. doi: 10.1093/bioinformatics/btad085 (PMC9940625; doi:10.1093/bioinformatics/btad085)
Supplement: btad085_Supplementary_Data [file btad085_supplementary_data.pdf]

Supplementary Information of "Multi-modal Representation Learning for Predicting Molecule-Disease Relation"

1 Supplementary Note: Architecture of the Feature Extractor

Feature Extractor We train a deep neural network to learn molecule multi-modal representation from molecule chemical structures represented by SMILES. In the SMILES, there are 64 unique tokens, e.g., "C", "=", etc., which are mapped to 64 integers accordingly. The feature extractor consists of an embedding layer, two 1-dimensional convolution layers, and a bi-directional GRUs layer. The embedding layer embeds each input integer to an 8-dimensional vector. The two 1-d convolution layers are with kernel sizes of 7 and 96 units. The bi-directional GRUs layer is with 128 hidden units with an "average" merge mode. The feature extractor is shared by indication and side effects.

Fusion Network The fusion network consists of two fully-connected (FC) layers. For both the input molecule representation and disease embedding, we first map them into 64-dimensional vectors separately using one FC layer. Instead of directly merging them, we find such a strategy works better as they are from different input spaces. Then they are fused using another FC layer into a 128-dimensional relation representation.

Classifier The classifier is composed of two fully-connected layers. The first layer consists of 48 units and the other is the output layer of 1 unit.

Discriminator The embedding discriminator is used to guide the feature extractor to map the "unmapped" novel molecules onto the same EHR embedding space and the relation discriminator is to encourage the predictor to generalize to novel molecule-disease combinations. Both consist of two fully-connected layers and one output layer, with 512, 512, and 1 units each layer for the embedding discriminator and 128, 128, and 1 for the relation discriminator.

Table 1: Comparison results evaluated in ROC-AUC of M2REMAP using different feature extractors for drug-indication prediction on PrimeKG [3] and side effects prediction on SIDER(Zhang) [4, 5].

Method	PrimeKG	SIDER 4.1
Transformer [1]	0.853	0.886
MPNN [2]	0.860	**0.907**
CNN+Bi-GRUs	**0.882**	0.901

Comparison of Feature Extractors We have studied different baseline feature extractors which include the proposed CNN+bi-GRUs and Transformer [1], both of which receive molecular SMILE, and MPNN [2] which works on molecular graphs. The results in Table 1 show that the proposed feature extractor achieves the best performance on PrimeKG [3] for predicting drug indications and attains comparable performance to that of MPNN on the SIDER(Zhang) [4, 5] for predicting drug side effects. For consistency, we use the proposed feature extractor across all experiments.

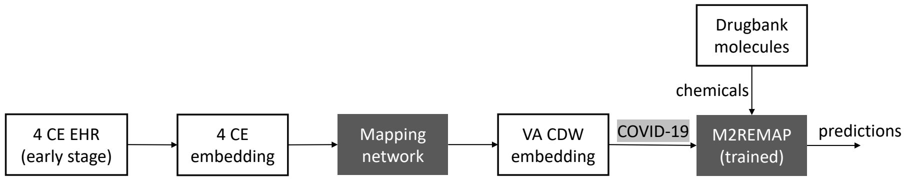

Figure 1: Pipeline of of M2REMAP to predict potential molecules for COVID-19. We first learn a new set of EHR embedding using 4CE data which includes concepts of COVID-19. Then, we transform 4CE embeddings to VA CDW embeddings and infer the relations between COVID-19 and Drugbank molecules.

2 Supplementary Note: Molecules for COVID-19

The pipeline to predict potential molecules for COVID-19 is illustrated in Figure 1. We first obtain a set of 200-dimensional embedding using the EHR data from the Consortium for Clinical Characterization of COVID-19 by EHR (4CE) Phase 2.2 [6]. It includes EHR data of COVID-19 patients from above 200 hospitals in 8 countries. Since the 4CE data is COVID-specific and contains only a small group of concepts and drugs, we map the 4CE embedding to the VA CDW embedding for relation inference. There are 2105 shared diagnostic concepts between 4CE and VA CDW EHR data. We train a multi-layer perception network (MLP) to learn the mapping from 4CE embedding to VA embedding via supervised

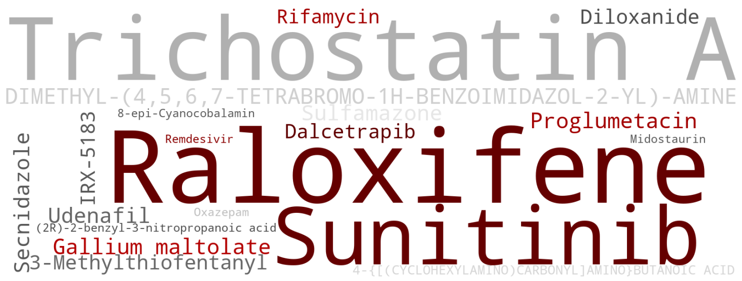

Figure 2: The top 20 molecules predicted for COVID-19 by M2REMAP (the red we find literature supports).

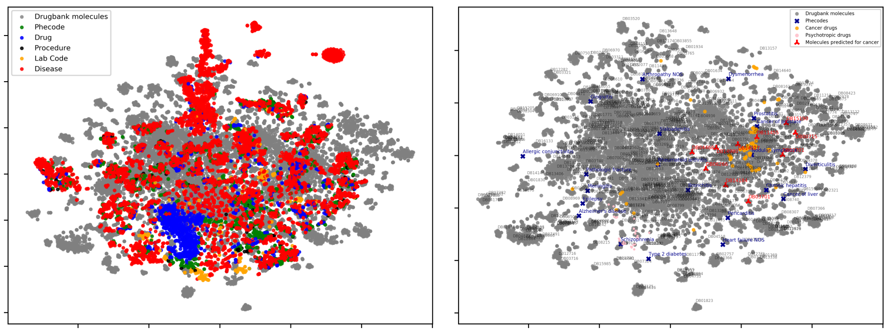

(a) Molecules and EHR concepts

(b) Molecules and diseases

Figure 3: Embedding visualization of all Drugbank molecules and clinical concepts. In (a), we show the predicted EHR embedding of Drugbank molecules. In (b) we visualize several typical diseases represented using Phecodes and the molecules that are literature-validated to be cancer-therapeutic.

regression using the 2105 shared codes as labels. We use the mean squared error as the training objective for the regression. The MLP regression network consists of two layers, namely a hidden layer with 200 units and an output layer with 100 units. In the 4CE, there are 5 concepts that are related to COVID-19, namely "PCR positive", "PCR negative", "U07.1", "COVID viral" and "COVID vaccine". Among them, we empirically find that "PCR positive" works better and thus represents COVID-19 using this concept. We use the M2REMAP trained on the annotated drug indications from PrimeKG [3] to predict the relations between COVID-19 and all Drugbank molecules [7].

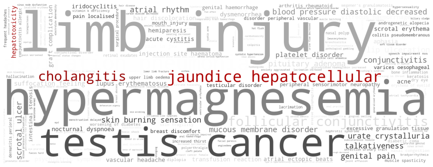

(a) DB06623 (Flupirtine)

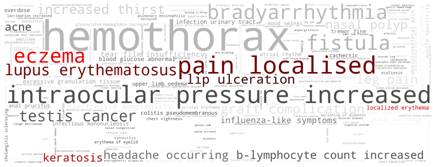

(b) DB13324 (Tetrazepam)

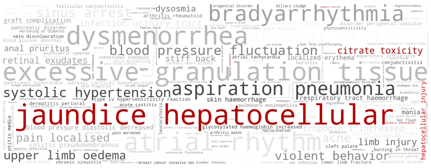

(c) DB01283 (Lumiracoxib)

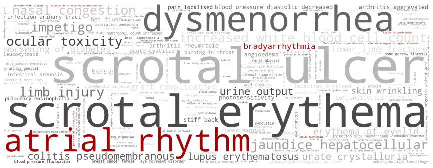

(d) DB09004 (Clobutinol)

Figure 4: Side effects predictions by M2REMAP of the 4 drugs that are withdrawn recently and have no reports in the SIDER 4.1 dataset [5] (the red are related to the cause of withdrawal).

3 Supplementary Note: Embedding Visualization of Novel Molecules

We visualize all EHR concepts and Drugbank molecules to show that M2REMAP successfully transforms novel molecule chemical structures to the EHR embedding space using the deep neural network. As shown in Figure 3 (a), the Drugbank molecules majorly follow the same embedding distribution as the clinical concepts. This facilitates M2REMAP to generalize to novel molecules to infer their relations with EHR diseases. Then, in Figure 3 (b), we visualize the molecules that are predicted to be therapeutic to cancers and are validated via literature reviews. Also, we visualize several representative diseases represented by diagnosis codes and 2% of the randomly selected Drugbank molecules. The results are consistent with the observations that molecules and the related indications tend to be close in the embedding space. For example, Vanoxerine (DB03701) is close to liver cancer and chronic hepatitis and is shown to treat hepatocellular carcinoma in [8].

4 Supplementary Note: Sampling of Negative Drug-Disease Relations

We select negative molecule-disease relation per the EHR embedding similarity. For each molecule, we require the selected negative side effects or indications to be dissimilar to any of the reported. Different threshold values are used for indications and side effects. For side effects, the threshold value is 0.2. For indications, the threshold value is 0.5.

5 Supplementary Note: Training Algorithm

The training details of the deep neural network to learn molecule-disease relations are provided in Algorithm 1.

Algorithm 1 Training the molecule-disease relation prediction network.

Require: D with EHR semantic embeddings, D_{label} with labeled drug-disease relations, D_{db} with Drugbank molecule chemicals, D_{mapped} containing the molecules with EHR embedding;

Ensure: Optimal $\mathbf{E}$, $\mathbf{M}$, $\mathbf{P}$, $\mathbf{D}$, $\mathbf{D}_{\mathsf{rela}}$;

$\boldsymbol{\theta}_{\mathbf{E}}$,$\boldsymbol{\theta}_{\mathbf{M}}$,$\boldsymbol{\theta}_{\mathbf{P}}$,$\boldsymbol{\theta}_{\mathbf{D}}$ $\leftarrow$ initialize network parameters;
repeat
  $\mathbf{x}_{un}$ $\leftarrow$ random mini-batch molecule from D_{db};
  $(\mathbf{x}_m, \mathbf{e}_m)$ $\leftarrow$ random mini-batch molecule-embedding pairs from D_{mapped};
  $(\mathbf{x}_l, \mathbf{e}_{d_l}, \mathbf{y}_l)$ $\leftarrow$ random mini-batch molecule-disease-label triplets from D_{label};
  $\mathbf{e}_{un}$ $\leftarrow$ random mini-batch semantic embedding from D;
  $\mathbf{e}_{d_{un}}$$\leftarrow$ random mini-batch disease embedding from D;
  $\mathcal{L} \leftarrow (\mathbf{M}(\mathbf{E}(\mathbf{x}_m)) - \mathbf{e}_m)^2$ //embedding loss of mapped molecules;
  $\mathcal{L}^{\mathsf{emb}}_{\mathsf{novel}} \leftarrow \log[\mathbf{D}(\mathbf{M}(\mathbf{E}(\mathbf{x}_{un})))] + \log[1 - \mathbf{D}(\mathbf{e}_{un})]$ //embedding loss of unmapped molecules;
  $\mathcal{L}^{\mathsf{rela}}_{\mathsf{novel}} \leftarrow \log[\mathbf{D}_{\mathsf{rela}}(\mathbf{P}(\mathbf{E}(\mathbf{x}_{un}), \mathbf{e}_{d_{un}}))] + \log[1 - \mathbf{D}_{\mathsf{rela}}(\mathbf{P}(\mathbf{E}(\mathbf{x}_l), \mathbf{e}_{d_l}))]$ //loss of novel molecule-disease pairs;
  $\leftarrow -\mathbf{y}_l * \log(\mathbf{P}(\mathbf{E}(\mathbf{x}_l), \mathbf{e}_{d_l})) - (1 - \mathbf{y}_l) * \log(1 - \mathbf{P}(\mathbf{E}(\mathbf{x}_l), \mathbf{e}_{d_l}))$ //prediction loss;
  // update parameters according to gradients;
  $\boldsymbol{\theta}_{\mathbf{E}} \xleftarrow{+} - \bigtriangledown_{\boldsymbol{\theta}_{\mathbf{E}}} + \beta\mathcal{L} + \gamma\mathcal{L}^{\mathsf{emb}}_{\mathsf{novel}}$ // update $\mathbf{E}$;
  $\boldsymbol{\theta}_{\mathbf{M}} \xleftarrow{+} - \bigtriangledown_{\boldsymbol{\theta}_{\mathbf{M}}} \mathcal{L}$ // update $\mathbf{M}$;
  $\boldsymbol{\theta}_{\mathbf{P}} \xleftarrow{+} - \bigtriangledown_{\boldsymbol{\theta}_{\mathbf{P}}} + \delta\mathcal{L}^{\mathsf{emb}}_{\mathsf{novel}}$ // update $\mathbf{P}$;
  $\boldsymbol{\theta}_{\mathbf{D}} \xleftarrow{+} - \bigtriangledown_{\boldsymbol{\theta}_{\mathbf{D}}} - \mathcal{L}^{\mathsf{emb}}_{\mathsf{novel}}$ // update $\mathbf{D}$;
  $\boldsymbol{\theta}_{\mathbf{D}_{\mathsf{rela}}} \xleftarrow{+} - \bigtriangledown_{\boldsymbol{\theta}_{\mathbf{D}_{\mathsf{rela}}}} - \mathcal{L}^{\mathsf{rela}}_{\mathsf{novel}}$ // update $\mathbf{D}_{\mathsf{rela}}$;
until deadline

6 Supplementary Note: Hyper-parameters Selection

We describe the selection of hyper-parameters β, γ, and δ, which balance the multiple objectives to train the model. We perform a grid search with 5-fold validations to find the selection. The values range from 0 to 1 with a span of 0.05. Finally, we get $\beta = 0.8$ for embedding learning of mapped molecules, $\gamma = 0.5$ for embedding learning of novel unmapped molecules, and $\delta = 0.1$ for relation learning of novel molecule-disease combinations. We aim to guide relation learning using the EHR semantic information. Thus, only the embedding learning

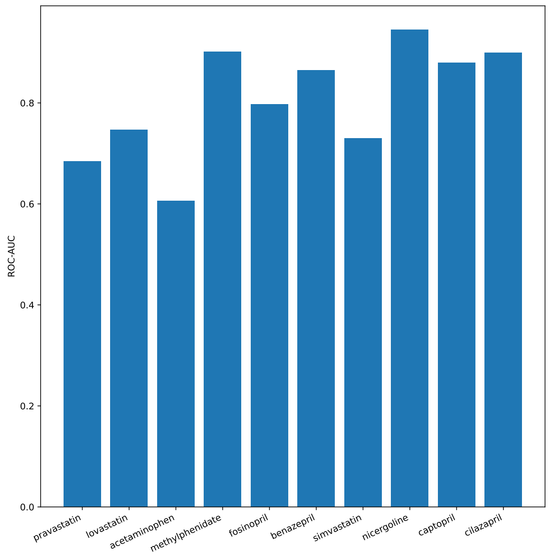

Figure 5: Prediction results of M2REMAP on the 10 drugs with annotations collected from *clinicaltrials*.

objective $\mathcal{L}_{\mathsf{emb}}$ is applied to the model when the training progress m, which increases from 0 to 1 as the training progresses, is smaller than 0.2. Further, we progressively increase the importance of adversarial generalization losses, $\mathcal{L}^{\mathsf{emb}}_{\mathsf{novel}}$ and $\mathcal{L}^{\mathsf{rela}}_{\mathsf{novel}}$, to prevent training instability. We set $\mathcal{L}^{\mathsf{emb}}_{\mathsf{novel}} = 0.5 * (\frac{2}{1+\exp(h \cdot m)} - 1)$ and $\mathcal{L}^{\mathsf{rela}}_{\mathsf{novel}} = 0.1 * (\frac{2}{1+\exp(h \cdot m)} - 1)$, where $h = -10$ and m denotes the training progress.

7 Supplementary Note: Side Effect Validation on *clinical-trials* Meta-analysis

To further evaluate the performance of side effect predictions, we manually create a small dataset of gold-standard labels on drug side effects, named SIDER-CT, based on literature reviews of clinical-trials meta-analysis results [9, 10, 11] for additional validations. It includes 257 negative drug-side-effect pairs and 103 positive pairs from 10 drugs.

We train M2REMAP on SIDER 4.1 with the 10 drugs removed and evaluate the performance by combining the reports from SIDER 4.1 and SIDER-CT. For each drug, the negative side effects are obtained only from the SIDER-CT while the positives are from both datasets. As shown in Figure 5, M2REMAP achieves a decent average ROC-AUC of 0.805.

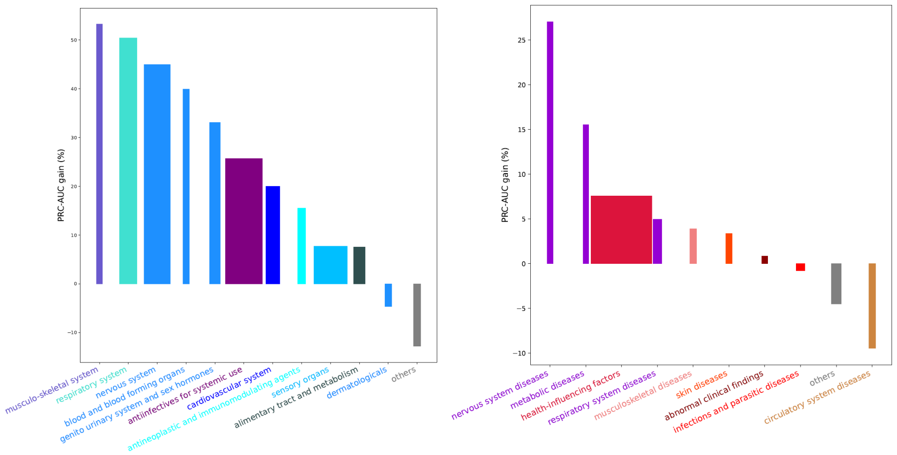

(a) Drugs

(b) Indications

Figure 6: Analysis of PRC-AUC gains brought by EHR semantic embedding in predicting drug indications. The bar width is proportional to the number of drugs/diseases contained in each group category.

8 Supplementary Note: Analysis of Improvements from EHR Semantic Embedding

We study how EHR semantic embedding vectors improve drug-disease predictions. We visualize the performance gain in PRC-AUC, namely $P_{\text{gain}} = P_{full}/(P_{\text{full}} - P_{\text{base}})$, where P_{full} denotes the PRC-AUC of the full model of M2REMAP that exploits semantic embedding vectors and P_{base} is the PRC-AUC of baseline model without EHR semantics. We visualize the performance gain by drug/disease groups. For drugs, we map them to RxCUI [1] and get their hierarchy. Each is mapped to the corresponding LEVEL1 concept, which consists of 14 groups such as "sensory organs", "respiratory system", etc. For indications/side effects, we map the CUIs to the ICD-10-CM, which includes 21 topics such as "neoplasms", "nervous-system diseases", etc. For each drug/disease group, we report the average PRC-AUC gain after introducing the EHR semantic embedding.

In Figure 6, we show the PRC-AUC gains in the drug indication prediction by performing 10-fold validations on the PrimeKG. For groups with less than 5 drugs/diseases observed, they are moved to an extra "others" group. 10 drug groups benefit from the introduction of EHR embedding and the top 3 are "musculo-skeletal system", "respiratory system", "nervous system". The 2 groups that suffer performance drops are "dermatologicals" and "others". Among the 10 indication disease groups observed, 8 of them benefit from the EHR embedding and the most significant are "nervous system diseases" and "metabolic diseases".

[1] https://mor.nlm.nih.gov/RxNav/

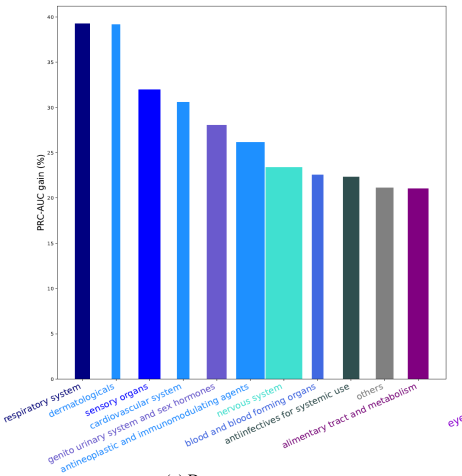

(a) Drugs

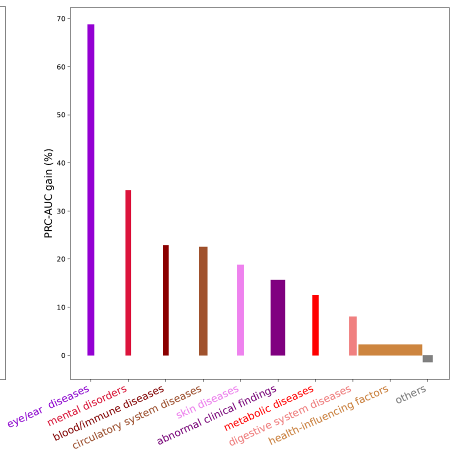

(b) Side effects

Figure 7: Analysis of PRC-AUC gains brought by EHR semantic embedding in predicting drug side effects. The bar width is proportional to the number of drugs/diseases contained in each group category.

3 diseases suffer performance drug, namely “circulatory system diseases”, “others”, “infections and parasitic diseases”.

In Figure 7, we show the PRC-AUC gains in the drug side effect prediction on the SIDER(Zhang) and report the results of the test drugs. For drug groups with less than 5 drugs and side effect groups with less than 10 side effects, they are moved to an extra “others” group. The 11 drug groups observed all benefit from the EHR embedding and the improvements from “respiratory system” and “dermatologicals” are the most significant. Among the 10 groups of side effects that are observed, 9 of them benefit from the EHR embedding. And the top group is “eye/ear diseases”, followed by “mental disorders” and “blood/immune diseases”. Only the “others” group slightly suffers a performance drop.

9 Supplementary Note: Sensitivity Analysis on the Dimensionality of Semantic Embedding

We perform sensitivity analysis on the dimensionality of embedding vectors. As shown in Tabel 2, the performances are comparable between the 50-dimensional and 100-dimensional EHR embedding vectors but become poorer as we increase the dimensions to 300 or 500. To be consistent, we use 100-dimensional embedding vectors across all experiments.

Table 2: Comparison results evaluated in PRC-AUC of M2REMAP using different dimensionality of semantic embedding vectors for drug-indication prediction on PrimeKG [3] and side effects prediction on SIDER(Zhang) [4, 5].

Dimensionality	PrimeKG	SIDER(Zhang)
500	**0.652**	0.510
100	0.649	**0.513**
300	0.636	0.505
500	0.627	0.497

References

[1] Ashish Vaswani, Noam Shazeer, Niki Parmar, Jakob Uszkoreit, Llion Jones, Aidan N Gomez, Łukasz Kaiser, and Illia Polosukhin. Attention is all you need. *Advances in neural information processing systems*, 30, 2017.

[2] Justin Gilmer, Samuel S Schoenholz, Patrick F Riley, Oriol Vinyals, and George E Dahl. Neural message passing for quantum chemistry. In *International conference on machine learning*, pages 1263–1272. PMLR, 2017.

[3] Payal Chandak, Kexin Huang, and Marinka Zitnik. Building a knowledge graph to enable precision medicine. *bioRxiv*, 2022.

[4] Wen Zhang, Feng Liu, Longqiang Luo, and Jingxia Zhang. Predicting drug side effects by multi-label learning and ensemble learning. *BMC bioinformatics*, 16(1):1–11, 2015.

[5] Michael Kuhn, Ivica Letunic, Lars Juhl Jensen, and Peer Bork. The sider database of drugs and side effects. *Nucleic acids research*, 44(D1):D1075–D1079, 2016.

[6] Jeffrey G Klann, Griffin M Weber, Hossein Estiri, Bertrand Moal, Paul Avillach, Chuan Hong, Victor Castro, Thomas Maulhardt, Amelia LM Tan, Alon Geva, et al. Validation of a derived international patient severity algorithm to support covid-19 analytics from electronic health record data. *medRxiv*, 2020.

[7] David S Wishart, Yannick D Feunang, An C Guo, Elvis J Lo, Ana Marcu, Jason R Grant, Tanvir Sajed, Daniel Johnson, Carin Li, Zinat Sayeeda, et al. Drugbank 5.0: a major update to the drugbank database for 2018. *Nucleic acids research*, 46(D1):D1074–D1082, 2018.

[8] Ying Zhu, Kun-Bin Ke, Zhong-Kun Xia, Hong-Jian Li, Rong Su, Chao Dong, Feng-Mei Zhou, Lin Wang, Rong Chen, Shi-Guo Wu, et al. Discovery of vanoxerine dihydrochloride as a cdk2/4/6 triple-inhibitor for the treatment of human hepatocellular carcinoma. *Molecular Medicine*, 27(1):1–14, 2021.

[9] Jayne E Edwards, Henry J McQuay, R Andrew Moore, and Sally L Collins. Reporting of adverse effects in clinical trials should be improved: lessons from acute postoperative pain. *Journal of pain and symptom management*, 18(6):427–437, 1999.

[10] Su Golder, Yoon K Loke, and Martin Bland. Meta-analyses of adverse effects data derived from randomised controlled trials as compared to observational studies: methodological overview. *PLoS medicine*, 8(5):e1001026, 2011.

[11] Mario Fioravanti, Taku Nakashima, Jun Xu, and Amit Garg. A systematic review and meta-analysis assessing adverse event profile and tolerability of nicergoline. *BMJ open*, 4(7):e005090, 2014.
